# Alleviation of Rheumatoid Arthritis by Inducing IDO Expression with *Trichinella spiralis* Recombinant Protein 43

**DOI:** 10.1155/2024/8816919

**Published:** 2024-01-17

**Authors:** Xiao Ma, Dongming Liu, Wenhao Yu, Caixia Han

**Affiliations:** ^1^College of Veterinary Medicine, Northeast Agricultural University, Harbin, China; ^2^Heilongjiang Key Laboratory of Zoonosis, Harbin, China

## Abstract

Rheumatoid arthritis (RA) represents the autoimmune disorder that shows aggressive arthritis as the main symptom. It is difficult to treat and can lead to joint deformation and function loss. At present, *Trichinella spiralis* (*T. spiralis*) antigen has attracted much attention because it plays a role in host immune regulatory mechanisms. Therefore, we selected *T. spiralis* recombinant protein 43 (Tsp43) to treat the bovine collagen type II (BCII)-induced mice RA model and explored its therapeutic mechanisms. This work first verified that Tsp43 could promote the expression of indoleamine 2, 3-dioxygenase (IDO) in dendritic cells (DCs) in vitro. Then, we randomized BALB/c mice (8 weeks old) into six groups, including control, phosphate buffer saline (PBS), BCII, BCII + heat inactivated Tsp43 (HiTsp43), BCII + Tsp43, and BCII + Tsp43 + 1-methyl-troptophan (1-MT) groups. To determine the therapeutic effect of Tsp43 on the BCII-induced mice RA model, relevant cytokines in each group and pathological changes in ankle joints were detected. To explore the mechanisms of Tsp43 on the BCII-induced mice RA model, we checked the expression of IDO in each group, CD4^+^T cell proliferation, and apoptosis. Collectively, Tsp43 decreased tumor necrosis factor-*α* (TNF-*α*) and interleukin-1*β* (IL-1*β*) expression in BCII-induced mice RA model and recovered the ankle injury to a certain extent. Tsp43 promoted high expression of IDO, caused expression of related apoptotic proteins in CD4^+^T cells, and caused apoptosis in CD4^+^T cells. In addition, Tsp43 reduced the proliferation of CD4^+^T cells. However, these effects can be inhibited by 1-MT (IDO inhibitor). These results suggested that Tsp43 played an important role in the treatment of arthritis by inhibiting the proliferation of CD4^+^T cells and inducing CD4^+^T cells apoptosis through the high expression of IDO. The purpose of this experiment was to provide a new idea for the treatment of RA and lay a foundation for the development of parasite-derived drugs for the treatment of RA.

## 1. Introduction

Rheumatoid arthritis (RA) represents the hereditary autoimmune disorder with the major features of persistent synovitis and articular cartilage damage [1]. Patients usually present with joint swelling and pain or even physical disability, which has seriously affected their lives. Rheumatoid factor (RF) is usually thought to be the cause of RA polyarthritis, but recently, studies have highlighted an important effect of abnormal T cell subsets on RA pathogenic mechanism [2–4]. Among them, the imbalance between CD4^+^T cells percentage and their function is an important factor leading to the development of RA [5]. During early RA, CD4^+^T cells are stimulated by exogenous and endogenous antigens and differentiate into diverse cell types that secrete various cytokines to promote the occurrence of RA inflammation [6]. After the imbalance of CD4^+^T cells in RA patients, their differentiation direction will be toward T helper cell 17 (Th17), where the secreted levels of interleukin-17 (IL-17), interleukin-21 (IL-21), and interleukin-23 (IL-23) will be significantly increased, which will stimulate the production of tumor necrosis factor-*α* (TNF-*α*), interleukin-1 (IL-1), interleukin-1 (IL-6), prostaglandin E2 (PGE2), and others by fibroblast-like synoviocytes, promote the production of osteoclasts and produce severe synovitis [7]. It has also been shown that CD4^+^T cells promote inflammation, bone erosion, and cartilage degradation by stimulating receptor activator of nuclear factor-*κ*B (NF-*κ*B) ligand expression and IL-17 production [8]. In conclusion, the imbalance of CD4^+^T cells is an important factor in the occurrence and development of RA.

The latest advances in the pharmacology and therapeutic methods of drugs for RA are based on the continuous development of drug development technologies, with the ultimate goal of relieving symptoms, slowing disease progression, and alleviating pathology [9]. Through a large number of clinical trials, some RA treatments have been approved for routine clinical practice. Among them, nonsteroidal anti-inflammatory drugs (NSNIDs) and corticosteroids are widely used to relieve pain, swelling, and reduce inflammation. However, some side effects cannot be ignored. In some patients, gastrointestinal bleeding, ulcers, headache, and osteoporosis will occur [10, 11]. Due to the adverse effects of nonsteroidal anti-inflammatory drugs and corticosteroids, disease-modifying antirheumatic drugs (DMARDs) have been developed as a type of immunosuppression used to prevent and reduce RA. Although DMARDs play a role in the efficacy of RA, it cannot be denied that treatment may be ineffective in some patients [12]. In general, restoring the immune balance of RA and awakening its immunosuppressive ability in vivo are still the main methods for the treatment of RA nowadays.

Indoleamine 2, 3-dioxygenases (IDO) is an enzyme expressed by DCs and is involved in tryptophan metabolism. It acts as the unique extrahepatic rate-limiting enzyme catalyzing tryptophan metabolism along the kynurenine metabolic pathway and can mediate the metabolism of more than 90% of tryptophan [13]. The change of IDO activity leads to a change in the concentration of tryptophan in the T cell microenvironment, damaging T cell immune function. CD4^+^T cells are the most sensitive to this change [14, 15]. Many studies have found that 1-methyltroptophan (1-MT) has a good inhibitory effect on IDO [16, 17]. Studies have shown that the excretory–secretory (ES) antigen of *Trichinella spiralis (T. spiralis*) can increase the expression of IDO and facilitate immune tolerance [18]. Among many proteins of ES antigen, a 43 kDa glycoprotein has been found, cloned, and expressed [19, 20]. The present work analyzed *T. spiralis* recombinant protein 43 (Tsp43)'s efficacy in the bovine collagen type II (BCII)-induced mice RA model. We explored the internal therapeutic mechanisms of Tsp43 on the BCII-induced mice RA model by checking the expression of IDO, CD4^+^T cell apoptosis, and proliferation. In this study, parasitic derivatives were introduced into the treatment of RA, and the therapeutic mechanisms were explored. The results indicated that the feasibility of treating RA with parasitic derivatives was confirmed.

In this study, the parasitic derivatives were introduced into the treatment of RA, and the therapeutic mechanism was explored to verify the feasibility of parasitic derivatives in the treatment of RA, aiming to provide a new idea for the treatment of RA and lay a foundation for the development of parasitic derivatives drugs for the treatment of RA.

## 2. Materials and Methods

### 2.1. Animals Used in Experiments

Eight-week-old healthy female BALB/c mice (SPF) provided by Liaoning Changsheng Biotechnology Co., Ltd., China, were used in experiments. The mice were adaptively fed for a 1-week period and were provided with food and water freely before starting the experiment. Each procedure was performed strictly following the care and use of laboratory animals released by China Health Research Institute. This work minimized the use of mice and minimized their pain.

### 2.2. Chemicals and Reagent Kits

The present study adopted the ImunoSep™ Mouse CD4^+^T Cell Enrichment Kit (PBM, Tianjin, China) for extracting CD4^+^T cells in mice spleen and stored at 4°C. Moreover, the apoptosis of CD4^+^T cells in mice spleen was analyzed by Annexin-V-FITC/PI Apoptosis detection kit (Meilunbio, Dalian, China).

### 2.3. Experimental Grouping

After 1 week of adaptive feeding, this work randomized 120 BALB/c mice into six groups, including control, phosphate buffer saline (PBS), BCII, BCII + heat inactivated Tsp43 (HiTsp43), BCII + Tsp43, and BCII + Tsp43 + 1-MT groups. RA was induced in four groups, except for the control group and PBS group, by punctuated injection of BCII into the tail root [21], whereas control group was not processed and those of PBS group were given normal phosphate buffer saline at equivalent volume. After successful induction of RA, HiTsp43 group received intraperitoneal injection of the emulsion of heat inactivated Tsp43 and adjuvant the BCII + Tsp43 group and the BCII + Tsp43 + 1-MT group were injected with the emulsion of Tsp43 and adjuvant by intraperitoneal injection at equivalent volume. The BCII + Tsp43 + 1-MT group was also given 1-MT for seven consecutive days.

### 2.4. Preparation of Tsp43

The Tsp43 recombinant protein has been successfully constructed and expressed in our laboratory. The frozen pET-32a-Tsp43 positive expression bacterial solution in the laboratory was revived, and the bacterial solution was broken by ultrasound. After purification by nickel column, it was dialyzed for renaturation. The endotoxin was removed by the ToxinEraser endotoxin removal kit and filtered. The concentration was measured by the bis-creatine (BCA) method. The samples were stored at −80°C. The purified protein was subjected to sodium dodecyl sulfate polyacrylamide gel electrophoresis (SDS-PAGE) separation, followed by staining with coomassie brilliant blue, and target protein band was identified.

### 2.5. Effect of Tsp43 on the Expression of IDO in DCs

The DCs in good condition were inoculated in a 12-well plate, cultured in a cell incubator for 12 hr to make them adhere to the wall, the common cell culture medium was changed to a cell culture medium containing Tsp43, and the control group was added with the same amount of phosphate buffered saline (PBS), and cultured in the cell incubator for 24 hr. After washing, fixation, membrane breaking, and blocking with PBS, IDO primary antibody (1 : 300 dilution) was added, followed by incubation in the dark for 12 hr at 4°C, and the primary antibody was washed three times by phosphate buffered saline with tween-20 (PBST). After incubation of the fluorescent secondary antibodies for 40 min, the secondary antibodies were collected, washed with PBST for three times, counterstained with 4′,6-diamidino-2-phenylindole (DAPI) for 5 min, and washed with PBS for three times. Finally, after 400 *μ*L PBS was added into each well, the expression of IDO in DCs was detected by fluorescence microscopy.

### 2.6. Selection of Tsp43 Dose

The mice in BCII + Tsp43 group were intraperitoneally injected with the doses of Tsp43 at 0/0.5/1/1.5/2/2.5/3 mg/kg, respectively. The total RNA in the spleen of the mice was extracted by the Trizol method and reversely transcribed into cDNA by using a reverse transcription kit. Based on the registration numbers of GenBank: mouse IDO (registration number: NM_008324), F: 5′-AGCAATCCCCACTGTATCCA-3′, R: 5′-GGTCCACAAAGTCACGCATC-3′, qRT-PCR was used to dynamically detect the expression changes of IDO mRNA in the protein group at different concentrations of Tsp43, so as to select the optimal dose of Tsp43.

### 2.7. Change of Serum Interleukin-1*β* (IL-1*β*) and TNF-*α* Content in Mice

The whole blood of mice was collected in vitro. The blood at room temperature was subject to 20-min natural coagulation as well as 20-min centrifugation (3,000 r/min). Serum was carefully aspirated. Enzyme-linked immunosorbent assay (ELISA) reagent kits were utilized to determine serum IL-1*β* and TNF-*α* contents.

### 2.8. Histopathological Examination of Mice Ankle Joints

This work immersed hind limbs of mice in each group into paraformaldehyde for a 24-hr period, followed by dehydration, dewaxing, and paraffin-embedding to obtain 4 *μ*m paraffin sections from the ankle. After dewaxing and alcohol gradient dehydration, the sections were later subject to hematoxylin and eosin (H&E) staining, while the light microscope was employed for observing ankle pathological alterations.

### 2.9. Western Blot of IDO Expression in Spleen

Mice splenic tissues were obtained in a sterile environment and prepared into single-cell suspensions that were dissolved in radio immunoprecipitation (RIPA) assay lysis buffer to extract protein. We measured total protein content with the BCA assay kit. Protein (about 50 *µ*g) was subject to boiling in a 5x SDS-PAGE sample loading buffer, followed by separation through 12% PAGE. Protein was imprinted on the nitrocellulose membrane. Later, a blocking buffer that contained 5% defatted milk was used to immerse membranes under ambient temperature for a 2-hr period. Subsequently, after overnight primary antibody incubation under 4°C, membranes were rinsed and probed using a peroxidase-labeled second antibody. Membranes were developed by a supersensitive chemiluminescent reagent (Sangon Biotech) and were exposed to a specific device. Densitometric analysis was performed for band quantification, while Image J software was employed for analysis.

### 2.10. Splenic CD4^+^T Cells Purification

After taking mice spleens, they were prepared in a single-cell suspension. ImunoSep™ Buffer was later added to resuspend cells at the 1 × 10^8^/mL cell density. An appropriate amount of single-cell suspension (not more than 2 × 10^8^ cells) was placed in a flow tube, and 20 *μ*L of Sorting Reagent A was added to every 100 *μ*L of single-cell suspension. After uniform mixing, it was incubated in a greenhouse for 10 min. The cells were washed with Imunosep™ Buffer and restored to the original volume, and 20 *μ*L Sorting Reagent B was added for every 100 *μ*L single cell suspension. After uniform mixing, it was incubated at room temperature for a 5-min period. Imunosep™ Buffer was added to dilute the sample to 2.5 mL. Flow tube was inserted into the magnetic pole, and the bottom of the flow tube passed through the magnetic pole channel until it touched the working table. After standing for 5 min, the liquid in the flow tube was carefully sucked out with a pipette without touching the wall of the tube. In total, 1 × 10^6^ cells were counted after centrifugation and resuspension. After adding 5 *μ*L antimouse CD4 FITC, samples were subject to 20-min incubation in dark under ambient temperature. Washing was done twice by PBS, then cells were resuspended into 500 *μ*L PBS. Purity was detected using flow cytometry.

### 2.11. CD4^+^T Cell Proliferative Capacity Detection

This work inoculated CD4^+^T cells (1 × 10^5^/well) extracted in spleen of each group into the 96-well plates, followed by culture within a cell incubator. After 0/6/12/24/48/76 hr, CCK-8 solution was added to incubate cells for a 2-hr period, and the optical density per well was determined at 450 nm.

### 2.12. Western Blot of CD4^+^T Cell

The CD4^+^T cell proteins were extracted and the total protein content was measured with the BCA assay kit. Protein (about 50 *µ*g) was subject to boiling in a 5x SDS-PAGE sample loading buffer, followed by separation through 12% PAGE. Protein was imprinted on the nitrocellulose membrane. Later, a blocking buffer that contained 5% defatted milk was used to immerse membranes under ambient temperature for a 2-hr period. After overnight primary antibody (anti-Bcl-2, anti-Bax, anti-cl-CASP9, anti-cl-CASP3, and anti-cl-PARP) incubation under 4°C, membranes were rinsed by PBST and probed using a peroxidase-labeled second antibody. Membranes were developed by a supersensitive ECL chemiluminescent reagent (Sangon Biotech) and were exposed to a specific device. Densitometric analysis was performed for band quantification, while Image J software was employed for analysis.

### 2.13. The Apoptotic CD4^+^T Cell Proportion of All Groups Was Detected by Flow Cytometry

The single-cell suspension of the mouse spleen was taken after removing red blood cells. In total, 1 × 10^6^ cells were added into each test tube, and 5 *μ*L antimouse CD4 APC was added. Incubation was done in the dark for 20 min after shaking and even mixing. Washing was done twice with PBS, followed by resuspension in 500 *μ*L PBS. After each tube was introduced with 20 *μ*L Annexin-V-FITC as well as 15 *μ*L PI, samples were subject to 20-min incubation in the dark and tested without washing.

### 2.14. Statistical Analysis

All data are presented as mean ± SD. Statistical analysis involved used GraphPad Prism 5. Image J software was used to quantify the band intensities. Differences between groups were assessed by one-way analysis of variance (ANOVA). *P* < 0.05 was considered statistically significant.

## 3. Results

### 3.1. Purification of Tsp43

**Figure 1:**
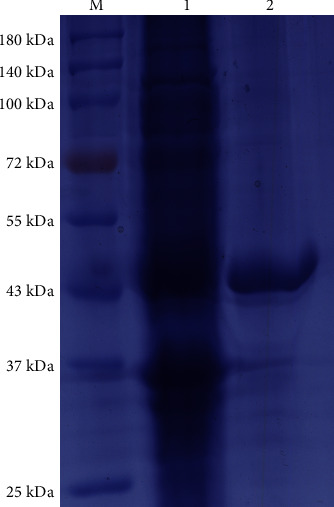
The unpurified bacterial protein and *Trichinella spiralis* recombinant protein 43 (Tsp43) purified by nickel column were stained with coomassie brilliant blue dye solution after SDS-PAGE. Lane M, protein marker; Lane 1, unpurified bacterial protein; and Lane 2, Tsp43.

The unpurified bacterial protein and Tsp43 purified by nickel column were stained with coomassie brilliant blue dye solution after SDS-PAGE, and the results are shown in Figure 1. Tsp43 showed a single target band at the correct position on the gel, which was consistent with the expected results.

### 3.2. Tsp43 Promotes IDO Expression in DCs

**Figure 2:**
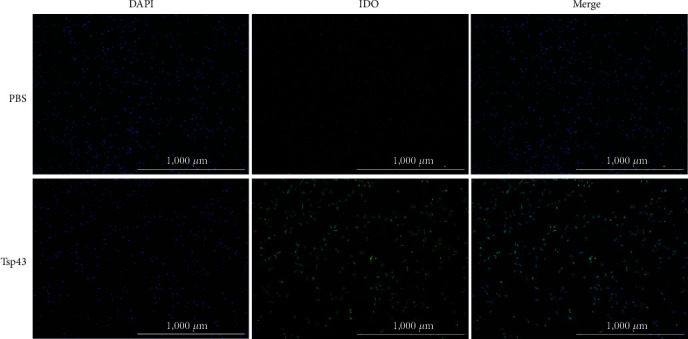
Effect of Tsp43 on expression of indoleamine 2, 3-dioxygenase (IDO) in dendritic cells (DCs) detected by immunofluorescence assay.

The green fluorescence in the PBS group was relatively weak, but it appeared in the field of view of DCs after 24 hr of exposure to Tsp43, indicating that Tsp43 could promote the expression of IDO in DCs (Figure 2).

### 3.3. Effects of Different Doses of Tsp43 on the Expression of Spleen IDO in BCII + Tsp43 Groups

**Figure 3:**
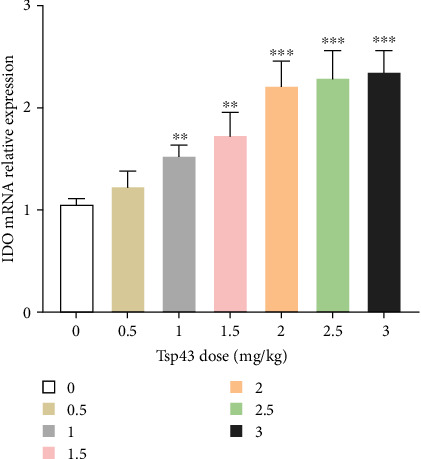
Effect of different doses of Tsp43 on the expression of IDO mRNA in mice spleen. Assays were performed in triplicate, and the data are presented as the mean ± SD.  ^*∗∗*^*P* < 0.01 compared with 0 group.  ^*∗∗∗*^*P* < 0.001 compared with control group.

We examined the effect of different intraperitoneal injection doses of Tsp43 on splenic IDO in mice (Figure 3). With the increase of the injection dose of Tsp43, the expression of IDO mRNA was also gradually increased. However, compared with the injection doses of 2.5 and 3 mg/kg, when the injection dose was 2 mg/kg, there was no more significant promotion effect on the expression of IDO mRNA. Therefore, the optimal injection dose of Tsp43 was 2 mg/kg.

### 3.4. Tsp43 Reduces Serum IL-1*β* and TNF-*α* Content

**Figure 4:**
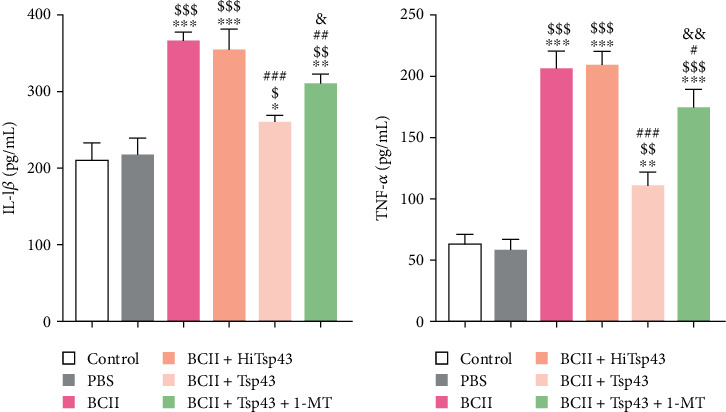
The contents of interleukin-1*β* (IL-1*β*) and tumor necrosis factor-*α* (TNF-*α*) in serum of mice in each group were detected by enzyme-linked immunosorbent assay (ELISA) kit. The contents of IL-1*β* in serum of mice in each group (a). The contents of TNF-*α* in serum of mice in each group (b). Assays were performed in triplicate, and the data are presented as the mean ± SD.  ^*∗*^*P* < 0.05,  ^*∗∗*^*P* < 0.01, and  ^*∗∗∗*^*P* < 0.001 compared with control group. ^$^*P* < 0.05, ^$$^*P* < 0.01, and ^$$$^*P* < 0.001 compared with PBS group. ^#^*P* < 0.05, ^##^*P* < 0.01, and ^###^*P* < 0.001 compared with BCII group. ^&^*P* < 0.05 and ^&&^*P* < 0.01 compared with BCII + Tsp43 group.

The contents of IL-1*β* (Figure 4(a)) and TNF-*α* (Figure 4(b)) were detected within mice whole blood serum by using the ELISA detection kit (Figure 4). There was no significant difference between control group and PBS group (*P*  > 0.05). The serum IL-1*β* and TNF-*α* contents of BCII group were markedly elevated (*P* < 0.001) relative to control, in line with the symptoms of RA [22, 23]. After the intervention treatment with Tsp43, the serum IL-1*β* and TNF-*α* of mice were significantly reduced (*P* < 0.001), while the contents of IL-1*β* and TNF-*α* were not changed by HiTsp43 (*P*  > 0.05), which indicated the potential therapeutic effects of Tsp43 on RA. After the 1-MT intervention, the serum IL-1*β* and TNF-*α* contents of mice were significantly increased again (*P* < 0.01). These findings indicated that 1-MT is a kind of IDO inhibitor and could attenuate the therapeutic effect of Tsp43 recombinant antigen on RA.

### 3.5. Tsp43 Suppressed RA Mice Histopathological Injury

**Figure 5:**
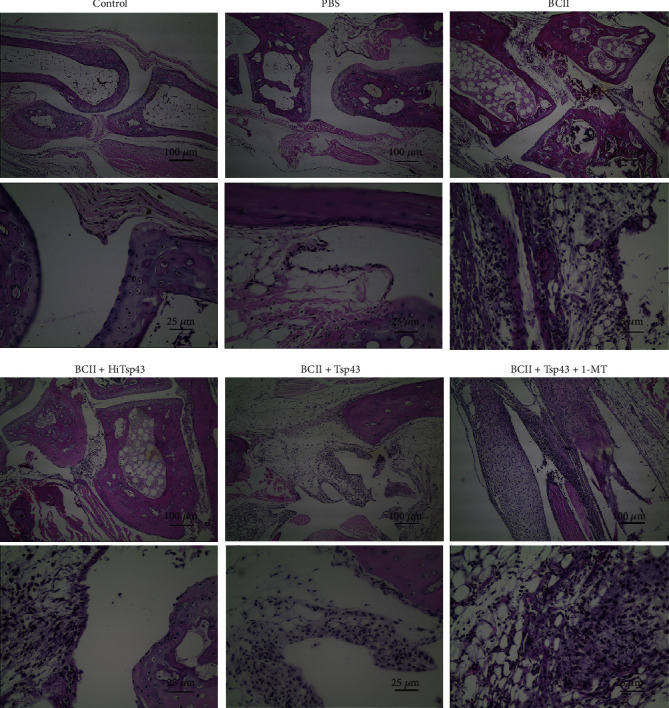
Hematoxylin and eosin (H&E) staining of ankle in hind limbs of mice. After decalcification, the ankle joints of the mice hind limbs were made into 4 *μ*m paraffin sections, and the ankle injuries were detected by H&E staining (original magnification: 100x, 400x).

In histology examination (Figure 5), for control group and PBS group, periosteal structure was found intact, the outer margin was tidy, and a clear and complete structure of the fibrous layer was seen. The osteoblasts were scattered in the bone plate, with oblate nuclei and abundant chromatin. The immature cells at the edge of the cartilage were flat and elliptical. The cells in the deep layer were large, round, and oval and existed in groups. The synovial fluid was dyed evenly, indicating that the synovial fluid was clear and transparent. In the BCII group and BCII + HiTsp43 group, bone destruction and obvious cartilage destruction were observed, together with the reduction of chondrocytes and the shedding of numerous cells at cartilage edge. Synovial interstitial edema and excessive T lymphocytes, plasma cells, macrophages, neutrophils, and other inflammatory cells infiltrated the interstitial and surrounding tissues. In the BCII + Tsp43 group, no significant cartilage damage was seen, the cells at the edge of the cartilage were slightly dropped, and the cell structure in the deep layer was complete. The synovial interstitium was slightly edematous with a small amount of inflammatory cell infiltration. In the BCII + Tsp43 + 1-MT group, cartilage destruction was significant, and a large number of cells at the cartilage edge fell off. Synovial interstitial edema was obvious, and infiltration of inflammatory cells could be observed within interstitial and surrounding tissues.

### 3.6. IDO Expression

**Figure 6:**
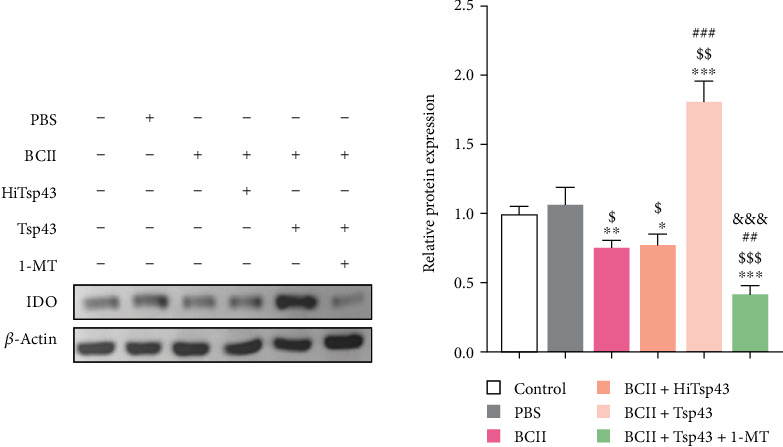
Western blot analysis the expression of IDO in spleen. Image J software was used to analyze the gray value. The data are presented as the mean ± SD.  ^*∗*^*P* < 0.05,  ^*∗∗*^*P* < 0.01, and  ^*∗∗∗*^*P* < 0.001 compared with control group.  ^$^*P* < 0.05,  ^$$^*P* < 0.01, and  ^$$$^*P* < 0.001 compared with PBS group.  ^##^*P* < 0.01 and  ^###^*P* < 0.001 compared with BCII group.  ^&&&^*P* < 0.01 compared with BCII + Tsp43 group.

The western blot results of IDO expression are shown in Figure 6. There was no significant difference between control group and PBS group (*P*  > 0.05). The expression of IDO significantly reduced (*P* < 0.01) in the BCII-induced mice RA model relative to control. Compared with BCII group, IDO level in BCII + Tsp43 group was significantly increased (*P* < 0.05), but the expression of IDO in BCII + HiTsp43 group was not statistically significant (*P* > 0.05), indicating that Tsp43 recombinant protein can effectively improve the expression of IDO. Additionally, the IDO expression in the BCII + Tsp43 + 1-MT group remarkably decreased (*P* < 0.001) relative to BCII + Tsp43 group, indicating that 1-MT was the effective inhibitor of IDO expression.

### 3.7. CD4^+^T Cell Purity

**Figure 7:**
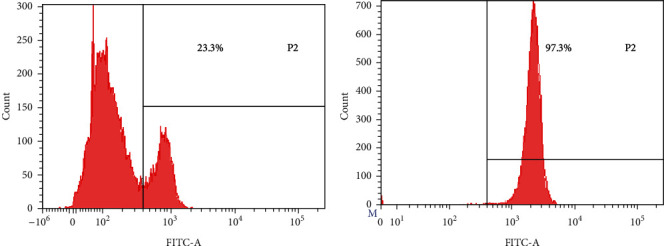
Purity of CD4^+^T cells before and after purification of splenic single cell suspension (CD4 FITC, the percentage represents the proportion of CD4^+^T cells to the total cells). Content of CD4^+^T cells in spleen of mice before purification (a). Purity of CD4^+^T cells purified by immunomagnetic bead method (b).

Splenic CD4^+^T cell purity is shown in Figure 7. As a result, CD4^+^T cells accounted for about 23% of the mice's spleen (Figure 7(a)). The purity was too low to meet the requirements for purity for subsequent experiments. The purity of CD4^+^T cells extracted by our CD4^+^T cell sorting kit was as high as 97% (Figure 7(b)), which could meet the needs of subsequent experiments.

### 3.8. Tsp43 Suppresses CD4^+^T Cell Proliferation

**Figure 8:**
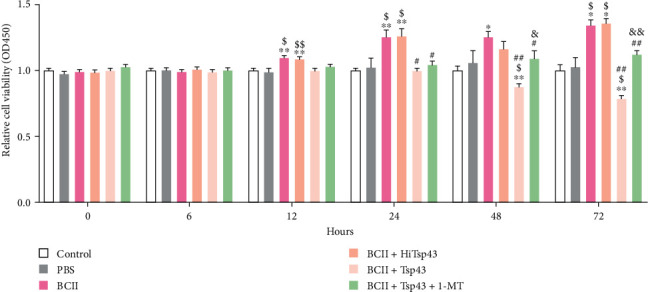
The CD4^+^T cells obtained by immunomagnetic bead method were cultured, and the change of proliferation ability was detected by CCK-8 kit at 0, 6, 12, 24, 48, and 72 hr. The data were analyzed from three independent experiments. The data were presented as the mean ± SD.  ^*∗*^*P* < 0.05 and  ^*∗∗*^*P* < 0.01 compared with control group. ^$^*P* < 0.05 and ^$$^*P* < 0.01 compared with PBS group. ^#^*P* < 0.05 and ^##^*P* < 0.01 compared with BCII group. ^&^*P* < 0.05 and ^&&^*P* < 0.01 compared with BCII + Tsp43 group.

After purification, this work tested CD4^+^T cells in each group for proliferation by CCK-8, as shown in Figure 8. CD4^+^T cells of different groups did not exhibit any obvious change between 0 and 6 hr. There was no significant difference in cell proliferation between the control group and PBS group (*P* > 0.05). Cell proliferation abilities in BCII group dramatically elevated (*P* < 0.05) at 12/24/48/72 hr relative to control, with obvious cell proliferation (*P* < 0.01) at 12/24 hr. The result indicated that the CD4^+^T cell proliferation ability of BCII-induced arthritis mice was high. Relative to BCII group, there was no statistical difference in BCII + HiTsp43 group but BCII + Tsp43 group showed significantly decreased (*P* < 0.05) cell proliferation at 24 hr and apparently declined (*P* < 0.01) proliferation at 48 hr and 72 hr. Collectively, Tsp43 decreased CD4^+^T cell proliferation in BCII-induced RA mice. Compared with the BCII + Tsp43 group, the BCII + Tsp43 + 1-MT group exhibited remarkably elevated (*P* < 0.05) cell proliferation at 48 hr, whereas apparently elevated (*P* < 0.01) cell proliferation at 72 hr. This finding suggested that 1-MT could inhibit the effect of Tsp43 on promoting CD4^+^T cell proliferation.

### 3.9. Tsp43 Promotes Apoptosis of CD4^+^T Cells

**Figure 9:**
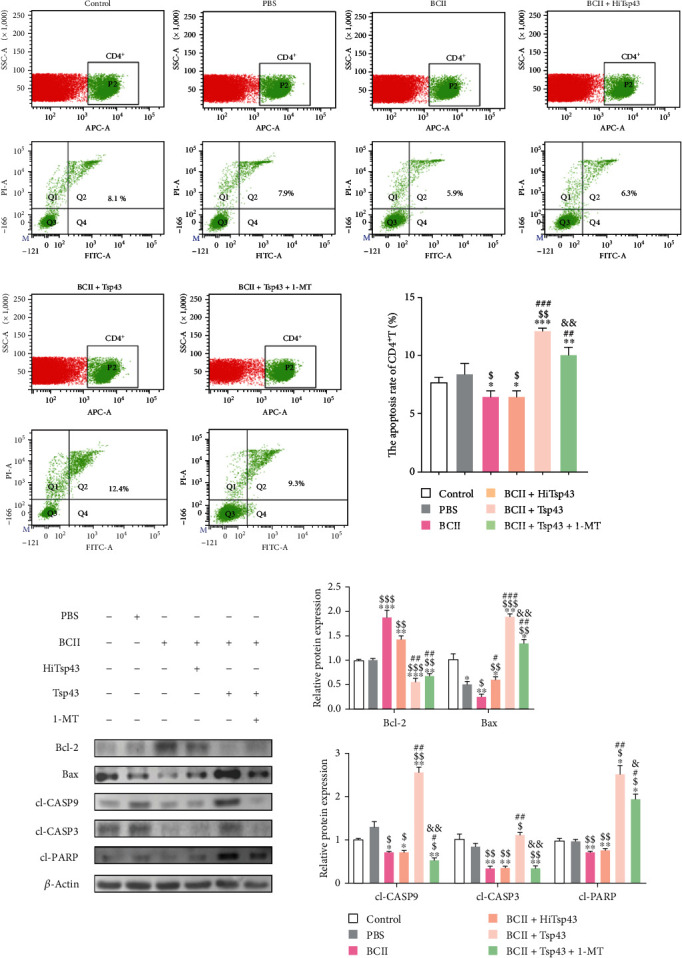
Apoptosis of CD4^+^T cells in spleen. Apoptosis of CD4^+^T in spleen was determined by staining with antimouse CD4 APC, Annexin V, and PI followed by flow cytometry (a). The total protein of CD4^+^T cells purified by immunomagnetic bead method was extracted from each group, and the expression of related apoptotic proteins was detected by Western blot and the gray values were analyzed by Image J software (b). All data are presented as the mean ± SD.  ^*∗*^*P* < 0.05,  ^*∗∗*^*P* < 0.01, and  ^*∗∗∗*^*P* < 0.001 compared with control group. ^$^*P* < 0.05, ^$$^*P* < 0.01, and ^$$$^*P* < 0.001 compared with PBS group. ^#^*P* < 0.05, ^##^*P* < 0.01, and ^###^*P* < 0.001 compared with BCII group. ^&^*P* < 0.05 and ^&&^*P* < 0.01 compared with BCII + Tsp43 group.

The splenic single-cell suspension of mice in each group was stained with antimouse CD4 APC and then subjected to apoptosis analysis using Annexin-V-FITC/PI cell apoptosis assay kit (Figure 9(a)). We also analyzed the expression of protein related to apoptosis in CD4^+^T cells (Figure 9(b)). There was no significant difference in CD4^+^T cell apoptosis rate between control group and PBS group. CD4^+^T cell apoptosis rate of BCII group was about 5.9% which was significantly lower (*P* < 0.05) than 8.1% of control group. Bcl-2 level of BCII group dramatically elevated (*P* < 0.001) relative to control group, and the level of Bax, cl-CASP9, cl-CASP3, and cl-PARP decreased (*P* < 0.01, *P* < 0.05, *P* < 0.01, *P* < 0.01). The apoptosis rate of CD4^+^T cells in BCII + HiTsp43 group was about 6.3%, and there was no significant difference relative to BCII group. CD4^+^T cell apoptotic rate of BCII + Tsp43 group was about 12.4%, which dramatically increased (*P* < 0.001) relative to BCII group. Bcl-2 level of BCII + Tsp43 group remarkably decreased (*P* < 0.001) relative to BCII group, and the level of Bax, cl-CASP9, cl-CASP3, cl-PARP dramatically elevated (*P* < 0.001, *P* < 0.01, *P* < 0.01, *P* < 0.01). The apoptotic rate of CD4^+^T cells in the BCII + Tsp43 + 1-MT group was approximately 9.3%, which apparently decreased (*P* < 0.01) compared with BCII + Tsp43 group. Bcl-2 level of BCII + Tsp43 + 1-MT dramatically elevated (*P* < 0.01) relative to BCII + Tsp43 group, and the level of Bax, cl-CASP9, cl-CASP3, and cl-PARP decreased (*P* < 0.01, *P* < 0.01, *P* < 0.01, and *P* < 0.05). The results showed reduced apoptosis of CD4^+^T cells in RA mice induced by BCII. Tsp43 could promote the apoptosis of CD4^+^T cells, and 1-MT inhibited the expression of IDO, which led to the reduction of apoptosis of CD4^+^T cells.

## 4. Discussion

RA represents the autoimmune disorder with the features of an imbalance of CD4^+^T cell number and function. CD4^+^T cells have an essential effect on RA and cause body to present a radical state of immune function [24, 25]. Recent studies have found that helminth infection can inhibit the occurrence of inflammatory bowel disease, diabetes, and asthma [26–28]. A member of worms, *T. spiralis*, and its secretions can also regulate the immune system and inhibit inflammation development [29, 30]. Disease-related applications for worms and their derivatives are evolving quickly.

TNF-*α* and IL-1*β* are important cytokines in the pathogenesis of RA. TNF-*α* can lead to an increase in the synthesis and secretion of chemokines as well as cell adhesion molecule levels, thereby accelerating recruitment of lymphocytes and monocytes to the inflammatory site [31]. IL-1*β* can activate chondrocytes and osteoclasts, leading to bone damage and bone resorption [32], and both of them can promote the apoptosis of chondrocytes [33]. In this study, we found that the BCII group had severe joint damage, and the content of TNF-*α* and IL-1*β* apparently increased relative to control. However, BCII + Tsp43 group had significantly reduced TNF-*α* and IL-1*β* levels and an improvement in ankle pathology compared with the BCII group and BCII + HiTsp43 group. Interestingly, after the 1-MT intervention, the BCII + Tsp43 + 1-MT group had a more severe ankle injury, and the TNF-*α* and IL-1*β* content was increased.

The high expression of IDO causes a large amount of tryptophan decomposition, induces T cell apoptosis, impairs the normal function of T cells, and inhibits T cell proliferation [34, 35]. According to our results, Tsp43 enhanced IDO expression. However, inhibition by 1-MT significantly decreased the expression of IDO in the BCII + Tsp43 + 1-MT group. Further investigating the changes brought by high expression of IDO, CD4^+^T cell apoptosis rate of BCII + Tsp43 group dramatically increased, and the apoptosis rate was decreased after 1-MT inhibition. We purified splenic CD4^+^T cells in each group and tested their proliferation. These findings showed a significantly higher proliferation of the BCII group and BCII + HiTsp43 group than that of the control group, with decreased proliferation of the BCII + Tsp43 group. However, the proliferation increased after the 1-MT intervention. This result indicates that the Tsp43 recombinant protein can promote the CD4^+^T cell apoptosis while reducing CD4^+^T cell proliferation, accompanied by high expression of IDO.

In this experiment, the RA mice model was induced by injection of BCII into the tail root. The histopathological and immunobiological aspects of these collagen-induced arthritis (CIA) mice are very close to human RA, and it is the most widely used and effective RA model at present. Studies have shown that inhibition of IDO activity or knockout of genes encoding IDO results in increased severity of CIA [36]. Moreover, research by Bianco et al. [37] has shown that DCs expressing IDO have immunosuppressive and anti-inflammatory effects and can reverse the established arthritis. Therefore, treatment of RA via the IDO pathway is a novel and effective means. It is well-known that the excessive activation of CD4^+^T cells in patients with RA leads to immune imbalance in the body. In this experiment, 1-MT (IDO inhibitor) was introduced and the original state of CD4^+^T cells in the body was kept by magnetic bead sorting, so as to accurately explore the practical effect of Tsp43 on the proliferation and apoptosis of CD4^+^T cells in RA mice through the IDO pathway. The results showed that Tsp43 inhibited the proliferation of CD4^+^T cells and promoted the apoptosis of CD4^+^T cells through the IDO pathway, thereby alleviating the immune imbalance caused by the excessive activation of CD4^+^T cells in RA mice. Similar studies have found that tryptophan deficiency caused by high expression of IDO inhibits T cell proliferation through GCN2 [38], and the products after high expression of IDO can cause T cell apoptosis [39]. Although we have not explored how Tsp43 affects CD4^+^T cell proliferation and apoptosis through the IDO pathway, this result is consistent with our experimental results. Although the condition of RA mice treated with Tsp43 was alleviated, other adverse reactions induced by Tsp43 were not deeply explored in this experiment, and the further research in this area will be gradually explored and improved in the future work.

## 5. Conclusion

This study showed that Tsp43 had a good therapeutic effect on RA mice, not only reducing the serum levels of IL-1*β* and TNF-*α* but also promoting the repair of ankle injury in RA mice. Further studies have shown that Tsp43 regulates the immune imbalance caused by overactivation of CD4^+^T cells in RA mice by inhibiting the proliferation of CD4^+^T cells and promoting the apoptosis of CD4^+^T cells with IDO.

Parasitic derivatives are rarely introduced into the treatment of RA. However, relevant studies have proved the positive effect of IDO in RA. This study has proved that Tsp43 can inhibit the proliferation of CD4^+^T cells in BCII-induced mice RA model and undergo apoptosis through IDO, thus exerting a certain therapeutic effect on RA, which has provided certain ideas for developing parasitic biological preparations as therapeutic means of RA. As the drug safety of Tsp43 has not been proved, relevant research on human subjects cannot be conducted at present. However, the histopathology and immunology of collagen-induced RA mice are close to human RA, which also implies that Tsp43 can be used in studies related to the treatment of human RA.

## Data Availability

All the data are included in this paper.
